# Investigation of the pathophysiology of the retina and choroid in Parkinson's disease by optical coherence tomography

**DOI:** 10.1007/s10792-021-02133-0

**Published:** 2021-12-02

**Authors:** Yasuaki Kamata, Naoto Hara, Tsukasa Satou, Takahiro Niida, Kazuo Mukuno

**Affiliations:** 1 Department of Orthoptics and Visual Sciences, School of Health Sciences, International University of Health and Welfare, 2600-1, Kitakanemaru, Ohtawara, Tochigi 324-8501 Japan; 2 Department of Ophthalmology, Yokohama Clinic of Kanagawa Dental University, Yokohama, Kanagawa Japan

**Keywords:** Choroid, Optical coherence tomography, Parkinson’s disease, Retina

## Abstract

**Purpose:**

The pathology of Parkinson's disease (PD) is suspected to affect the retina and choroid. We investigated changes in the retina and choroid of patients with PD using optical coherence tomography.

**Methods:**

We examined 14 patients with PD and 22 patients without PD. Patients without PD had no ophthalmic disease other than cataracts. In addition, it was also confirmed that there was no neurodegenerative disease. The retinal nerve fiber layer, ganglion cell layer + inner plexiform layer, and choroidal thickness were compared between both groups. Additionally, the choroidal image was divided into the choroid area, luminal area, and interstitial area using the binarization method, and the area of each region and the percentage of luminal area in the choroid area were analyzed.

**Results:**

Patients with PD had a significantly thinner ganglion cell layer + inner plexiform layer compared to those without PD. The choroid area, luminal area, and interstitial area were significantly decreased in patients with PD compared to those without PD. Seven patients with PD who were successfully followed up showed decreased retinal nerve fiber layer and interstitial area after 3 years.

**Conclusion:**

Autonomic nervous disorders and neurodegeneration in PD can cause thinning of the retina and choroid, as well as a reduction in the choroid area.

## Introduction

Parkinson's disease (PD) is a central neurodegenerative disease that involves the degeneration of dopaminergic cells in the substantia nigra of the midbrain, the reduction in dopamine activity in the striatum, and the relative potentiation of acetylcholine. PD causes various ocular symptoms (e.g., dry eye and cataracts) as well as abnormalities of pupils affected by autonomic nervous function [1, 2]. Adam et al. [3] reported that neurodegeneration in patients with PD causes retinal thinning.

Evaluation of the retina using optical coherence tomography (OCT) has been suggested as a potential biomarker for PD [4]. OCT constructs images using the signal strength of the light reflected from the eye tissue, generated by administering infrared irradiation to the patient's eye. OCT can be used to noninvasively acquire cross-sectional and three-dimensional images of the eye in a short time. Thus, it is used in current ophthalmic practice to assess qualitative and quantitative changes in ocular diseases. Recently, swept-source OCT (SS-OCT), which uses a wavelength (approximately 1050 nm) that enables superior depth penetration into the tissue compared with the light wavelength of conventional OCT (approximately 840 nm), has been developed. Therefore, it can be used to construct high-quality cross-sectional images from the vitreous to the choroid. Choroidal imaging using OCT has also attracted attention for the observation of neurological diseases [5].

The choroid, located between the retina and sclera of the eye, is a brown membranous tissue that comprises a large proportion of the ocular blood flow. Nickla and Wallman [6] reported that changes in choroidal blood flow were also affected by autonomic nervous function. The spaces between the choroidal vessels contain fibroblasts, melanocytes, mast cells, and plasma cells. The melanocyte content in the choroid is approximately 60% [7]. Choroidal melanocytes protect the eyes by blocking and absorbing external light [8]. Recently, a relationship between PD and melanocyte-derived malignant melanoma has been reported [9], and the observation of the choroid may enable the early detection of both diseases. However, the effects of autonomic nervous system disorders on the choroid remain unknown. In this study, we scanned the retina and choroid of patients with PD using SS-OCT to examine how these tissues are affected by autonomic dysfunction in PD.

## Methods

### Participants

The procedures used in this study were approved by the Institutional Review Board of the International University of Health and Welfare Hospital (approval number 13-B-237) and conformed to the tenets of the Declaration of Helsinki.

We investigated the thickness and choroidal area of the retina and choroid in patients with and without PD who visited the International University of Health and Welfare Hospital. Fourteen patients with PD and 22 without PD were included. Patients without PD were used as controls, and the area and thickness of their retina and choroid were compared with those of patients with PD. The eyes were randomly selected from all patients. All patients underwent an extensive ophthalmologic examination, including refractive error, axial length, intraocular pressure, slit-lamp examination of the anterior segment, and SS-OCT. Patients with PD were evaluated using the Movement Disorder Society-sponsored revision of the Unified Parkinson’s Disease Rating Scale for Parkinson’s disease, which assesses disease severity using the Hoehn and Yahr scale. We confirmed the presence of autonomic dysfunction (e.g., bradykinesia, resting tremor, and postural instability) in all patients with PD. All patients with PD were treated with levodopa using Levodopa/Carbidopa or Levodopa/Benserazide (mean dose, 550 mg daily; range, 300–900 mg). Patients without PD had no ophthalmic disease other than cataracts. In addition, it was also confirmed that there was no neurodegenerative disease. To minimize the effect of variations in circadian rhythm, we examined the patients between 9 a.m. and 11 a.m.

Second, we compared changes in the retina and choroid at the first visit and after 3 years in patients with PD. These participants comprised seven patients with PD in whom we were able to perform OCT 3 years after their first visit.

### Measurement of retina and choroid by SS-OCT

**Fig. 1:**
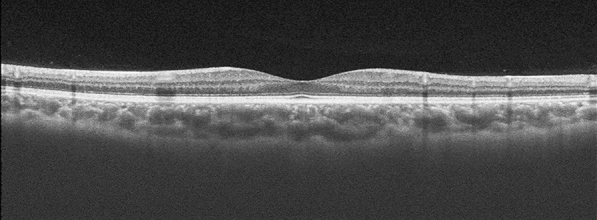
Cross-sectional images of the choroid and retina obtained by swept- source optical coherence tomography

**Fig. 2:**
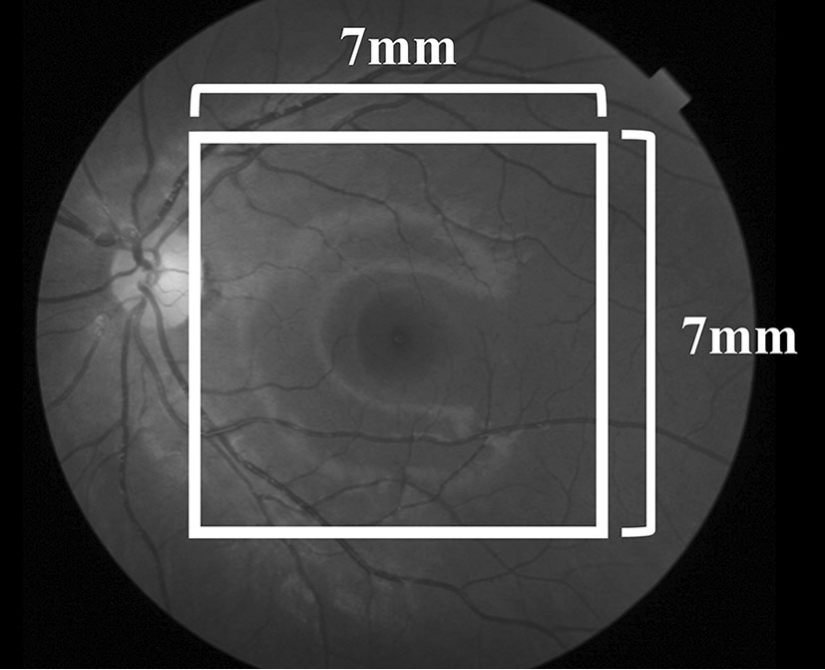
Measurement range for swept- source optical coherence tomography, shown in a fundus image

**Fig. 3:**
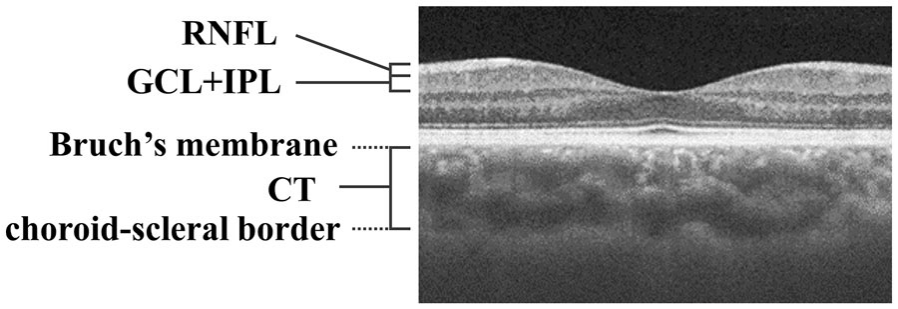
Enlarged image of the macula from Fig. 1. The layer above the retina, characterized by high brightness, is the retinal nerve fiber layer (RNFL), while the underlying layer is the ganglion cell layer + inner plexiform layer (GCL + IPL). Choroidal thickness (CT) was defined as the distance from Bruch’s membrane to the choroid-scleral border

Images of the retina and choroid were obtained using SS-OCT (Topcon Corp., Tokyo, Japan), and three-dimensional macular scanning was performed (3D-Macular 7 × 7 mm) (Figs. 1 and 2). The images were quantitatively analyzed using the automatic segmentation algorithm of SS-OCT. We measured the retinal nerve fiber layer (RNFL) thickness, ganglion cell layer + inner plexiform layer (GCL + IPL) thickness, and choroidal thickness (CT). The RNFL thickness and GCL + IPL thickness were analyzed based on the total thickness. CT was measured from Bruch’s membrane to the choroid–scleral border (Fig. 3).

### Binarization of choroidal SS-OCT images

**Fig. 4:**
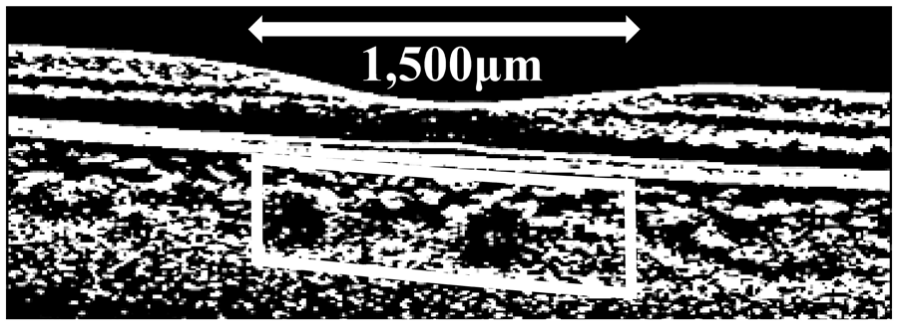
Optical coherence tomography image subjected to binarization using ImageJ software (US National Institutes of Health, Bethesda, MD, USA). After binarization, the vascular region is black (luminal area), and the interstitial region is white (interstitial area); these can be used to calculate the area of each choroid region. The measurement range was calculated as a 1,500-μm width in the subfoveal region (i.e., 750 μm temporally and 750 μm nasally from the fovea), and its length extended from Bruch’s membrane to the choroid–scleral border

After recording the SS-OCT images, the choroid area (C) was calculated using ImageJ software (National Institutes of Health, Bethesda, MD, USA) [10, 11]. C was defined with a width of 1500 μm in the subfoveal region (i.e., 750 μm temporally and 750 μm nasally from the fovea) and a length from Bruch’s membrane to the choroid–scleral border. Moreover, in this method, an OCT image is subjected to binarization processing, and the area can be obtained by dividing the choroid into vascular and interstitial regions. In an OCT image of the choroid, the vascular region is depicted with low luminance, and the interstitial region is depicted with high luminance. After binarization is performed on the OCT image, the vascular and interstitial regions are visible as black and white, respectively; thus, the area of each region can be calculated. Binarization was performed using the Niblack method [10]. After binarization, the vessel area was defined as the luminal area (L), the remaining area was defined as the interstitial area (I), and the percentage of L in C (L/C) was calculated (Fig. 4).

### Statistical analysis

All parameters of the retina and choroid were compared in patients with and without PD using the Mann–Whitney U test, and the Wilcoxon signed-rank test was used to assess these parameters in patients with PD at their first visit and after 3 years. The threshold for statistical significance was set at *p* < 0.05.

## Results

### Comparison of the retina and choroid in patients with and without PD

Demographic data of all participants are shown in Table 1. Age, sex, and refractive error were not significantly different between the two groups. All patients with PD exhibited stage III disease severity according to the Hoehn and Yahr scale.

**Table 1:** Demographic data

	Patients with Parkinson's disease (*n* = 14)	Patients without Parkinson's disease (*n* = 22)	*p*-value
Age (years)	77.4 ± 7.2	72.9 ± 7.8	0.1160
Females, n (%)	11 (78.6)	15 (68.2)	0.4975
Refractive error (D)	0.08 ± 2.25	− 0.82 ± 1.09	0.1673
Axial length (mm)	22.95 ± 6.27	23.47 ± 1.16	0.0717
Disease duration (months)	127.0 ± 62.7	–	–

The results showed that the RNFL thickness, GCL + IPL thickness, and CT were 37.6 ± 7.6 μm, 57.8 ± 3.7 μm, and 154.1 ± 53.3 μm in patients with PD and 38.0 ± 5.4 μm, 61.9 ± 5.3 μm, and 219.9 ± 65.3 μm in those without PD, respectively. The GCL + IPL and choroid were thinner in patients with PD than in those without PD (*p* < 0.05). Conversely, no significant difference was observed in the RNFL thickness between the groups (Table 2).

**Table 2:** Thicknesses of the retinal nerve fiber layer, ganglion cell layer, and inner plexiform layer and choroid

	Patients with Parkinson's disease (*n* = 14)	Patients without Parkinson's disease (*n* = 22)	*p*-value
Retinal nerve fiber layer (RNFL) thickness (μm)	37.6 ± 7.6	38.0 ± 5.4	0.6960
Ganglion cell layer + inner plexiform layer (GCL + IPL) thickness (μm)	57.8 ± 3.7	61.9 ± 5.3	0.0102*
Choroidal thickness (CT) (μm)	154.1 ± 53.3	219.9 ± 65.3	0.0041**

*p < 0.05; **p < 0.01

**Fig. 5:**
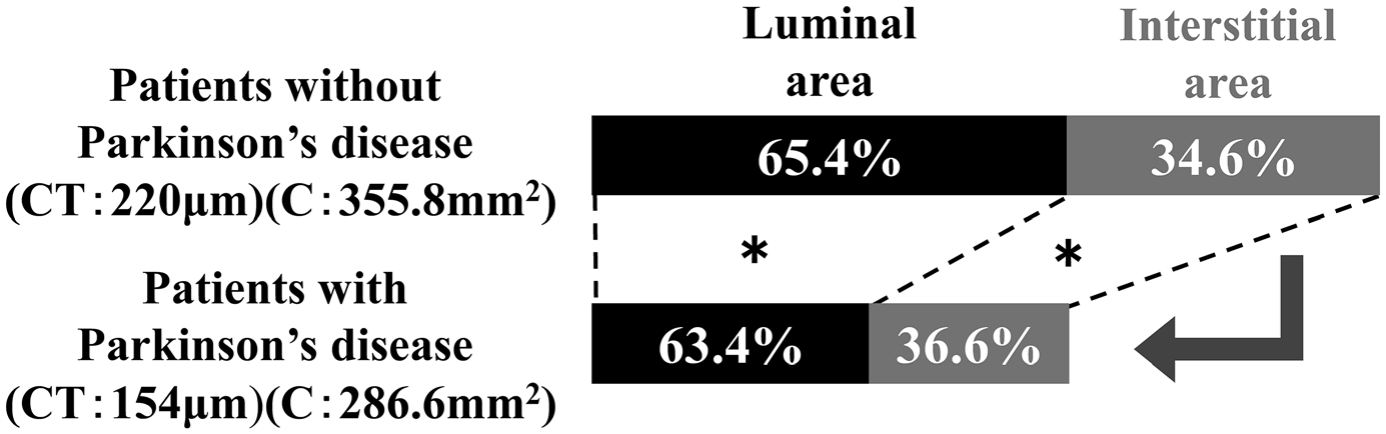
Percentage of the luminal area (L) in the choroidal area (C) in patients with and without Parkinson’s disease (PD). Both areas were decreased in PD, while maintaining the ratio. The choroid was thinner in patients with PD (154 μm) than in those without PD (220 μm). C was reduced in patients with PD (355.8 mm^2^) compared with those without PD (286.6 mm^2^)

The choroidal OCT images binarization results showed that the C, L, I, and L/C were 286.6 ± 67.4 mm^2^, 183.3 ± 51.0 mm^2^, 103.3 ± 18.5 mm^2^, and 63.4 ± 3.7% in patients with PD and 355.8 ± 82.9 mm^2^, 234.4 ± 63.7 mm^2^, 121.3 ± 21.6 mm^2^, and 65.4 ± 3.2% in those without PD, respectively. C, L, and I were lower in patients with PD than in those without PD (*p* < 0.05). L/C was not significantly different between the two groups (Table 3). Since the L/C ratio was not significantly affected in patients with PD, both regions showed less thickness and considerably thinner CT compared to those without PD (Fig. 5).

**Table 3:** Binarization processing of the choroid

	Patients with Parkinson's disease (*n* = 14)	Patients without Parkinson's disease (*n* = 22)	*p*-value
Choroidal area (C) (mm^2^)	286.6 ± 67.4	355.8 ± 82.9	0.0137*
Luminal area (L) (mm^2^)	183.3 ± 51.0	234.4 ± 63.7	0.0231*
Interstitial area (I) (mm^2^)	103.3 ± 18.5	121.3 ± 21.6	0.0149*
Percentage of L in C (L/C) (%)	63.4 ± 3.7	65.4 ± 3.2	0.1118

*p < 0.05

### Comparison of the retina and choroid during the first visit and after 3 years in patients with PD

We performed SS-OCT in 7 patients with PD (one male, six females), 3 years after their first visit. Two of these patients progressed to stage IV of the Hoehn and Yahr scale.

The results showed that the RNFL thickness, GCL + IPL thickness, and CT were 35.1 ± 8.0 μm, 56.7 ± 3.1 μm, and 153.1 ± 62.8 μm, respectively, during the first visit, and 33.3 ± 7.5 μm, 55.1 ± 3.0 μm, and 151.7 ± 61.0 μm, respectively, after 3 years. The RNFL thickness after 3 years was decreased compared to that at the first visit (*p* < 0.05). The GPL + IPL thickness and CT were not significantly different after 3 years (Table 4).

**Table 4:** Thicknesses of the retinal nerve fiber layer, ganglion cell layer, and inner plexiform layer and choroid in seven patients with Parkinson's disease

	First visit	At 3-year follow-up	*p*-value
Retinal nerve fiber layer (RNFL) thickness (μm)	35.1 ± 8.0	33.3 ± 7.5	0.0431*
Ganglion cell layer + inner plexiform layer (GCL + IPL) thickness (μm)	56.7 ± 3.1	55.1 ± 3.0	0.0679
Choroidal thickness (CT) (μm)	153.1 ± 62.8	151.7 ± 61.0	0.6858

*p < 0.05

The results of binarization of the choroidal OCT images showed that the C, L, I, and L/C were 275.3 ± 80.4 mm^2^, 177.9 ± 60.7 mm^2^, 97.5 ± 20.7 mm^2^, and 63.9 ± 3.7%, respectively, at the first visit, and 245.5 ± 89.3 mm^2^, 161.7 ± 58.5 mm^2^, 83.8 ± 31.3 mm^2^, and 66.0 ± 2.0%, respectively, after 3 years. After 3 years, I decreased compared to that at the first visit (*p* < 0.05), but the C, L, and L/C ratios were not significantly different (Table 5). Table 6 shows the changes in the retina and choroid parameters 3 years after the first visit in each patient with PD.

**Table 5:** Binarization processing of the choroid in seven patients with Parkinson's disease

	First visit	At 3-year follow-up	*p*-value
Choroidal area (C) (mm^2^)	275.3 ± 80.4	245.5 ± 89.3	0.0630
Luminal area (L) (mm^2^)	177.9 ± 60.7	161.7 ± 58.5	0.1763
Interstitial area (I) (mm^2^)	97.5 ± 20.7	83.8 ± 31.3	0.0425*
Percentage of L in C (L/C) (%)	63.9 ± 3.7	66.0 ± 2.0	0.2367

**Table 6:** PD duration and the amount of change in retinal and choroidal parameters 3 years after the first visit in each patient

Disease duration (month)	RNFL (μm)	GCL + IPL (μm)	CT (μm)	C (mm^2^)	L (mm^2^)	I (mm^2^)
228	− 1	0	− 32	− 67.7	− 58.4	− 9.3
178	− 5	− 5	15	1.8	2.2	− 0.3
156	− 3	− 1	16	− 17.9	− 5.5	− 12.5
109	− 2	− 3	0	− 31.5	− 13.8	− 17.7
84	0	− 2	0	8.3	0.9	7.3
84	− 2	0	− 30	− 87.3	− 44.2	− 43.2
50	0	0	21	− 14.7	5.4	− 20.1

C choroid area; CT choroidal thickness; GCL + IPL ganglion cell layer and inner plexiform layer; I interstitial area; L luminal area; PD Parkinson's disease; RNFL retinal nerve fiber layer

## Discussion

In this study, we used SS-OCT to investigate the effects of autonomic dysfunction and neurodegeneration in PD on the retina and choroid. Our results revealed that the GCL + IPL and CT were thinner, and C, L, and I were decreased in patients with PD compared to those without PD. A 3-year follow-up of patients with PD revealed a decreased RNFL thickness and I.

The source of retinal thinning may be related to neurodegeneration associated with the pathology of PD. The appearance of Lewy bodies, which contain abnormal deposition of amyloid fibrils, can be observed in the nerve cells of patients with PD. Such amyloidosis also occurs in the GCL of ocular tissues and may cause nerve degeneration in the retina [12]. Furthermore, retinal dopaminergic neurons are present in amacrine cells, and the IPL corresponds to the dendrites of amacrine cells. It has been reported that visual pathologies in patients with PD may be caused by dopamine reduction in retinal amacrine cells [13, 14]. The concentration of retinal dopaminergic neurons in patients with PD has been shown to be lower than that in healthy individuals [15]. The reduction in dopamine caused by PD results in the thinning of the IPL due to a deficit in retinal dopaminergic neurons [3, 16]. Therefore, PD-induced neurodegeneration may be associated with GCL and IPL thinning in the macular region, as observed in this study.

Contrastingly, the RNFL thickness in patients with PD was not significantly different from that in patients without PD in this study. Kirbas et al. [17] and Turgay et al. [14] reported that the RNFL and GCL + IPL were thinner around the optic disc in patients with PD than in healthy controls. In this study, the RNFL of the macular region was considered; the discrepancy between our findings and theirs suggests that to investigate minor changes in RNFL thickness, analysis of the region surrounding the optic disc may be more appropriate. However, Altıntaş et al. [18] reported that the RNFL thickness was decreased in patients with PD than in healthy controls. In our study, macular analysis showed that the retinas of patients with PD were significantly thinner after 3 years compared to the first visit. Atum et al. [19] followed up the retina in the macular analysis of patients with PD for 2 years. As a result, a thinner RNFL thickness was reported in patients with PD than in healthy controls. Therefore, it may be useful to combine macular and optic disc analyses results to analyze the retina of patients with PD. Similarly, glaucoma is an eye disease that causes thinning of the RNFL and GCL + IPL. Patients with PD have been shown to have a high prevalence of glaucoma [2], and measurement of the retina using OCT may be important for both observations.

In this study, the choroid was thinner in patients with PD compared to patients without PD; thinning of the choroid was observed as C decreased, and both L and I decreased concomitantly. We suspect that there was no significant difference in L/C because C decreased while the relative proportions of L and I remained constant. It has been reported that the CT in patients with PD is less than that of healthy individuals, consistent with the current findings [20, 21]. The subjects in this study were non-myopic based on the refractive error and axial length results. Therefore, the effect of choroidal thinning was considered small. We performed binarization to investigate the cause of the choroid thinning. Few reports have examined the choroid of patients with PD using this method.

Considering that choroidal thinning is caused by a reduction in L, the reduced blood flow may be due to autonomic dysfunction in PD. The smooth muscle of the choroidal vascular wall contains autonomic innervation, which is associated with changes in ocular blood flow [6]. Choroidal blood flow exhibits a circadian rhythm [22]; thus, autonomic dysfunction in PD may affect choroidal blood flow. Therefore, the reduction in L observed in this study may be due to the reduced blood flow associated with chronic circulatory disturbances caused by autonomic dysfunction in PD.

The decrease in I may be induced by changes in the number of melanocytes in the interstitial region. In the pathology of PD, the black tone of the substantia nigra is reduced due to the loss of cells with melanin. Melanocytes are produced when tyrosine (a precursor of dopamine) is exposed to ultraviolet light and then activated by tyrosinase. Tyrosine is a precursor of the neurotransmitter dopamine. In PD, where dopamine is selectively impaired, tyrosinase activity and melanocyte production may be concomitantly reduced. Changes in the choroid of patients with PD may progress more rapidly than changes in the choroid of patients of the same age without PD, and the degeneration of melanocytes may be involved in the thinning of the choroid. Additionally, the choroid of patients with PD after 3 years showed a decrease in I only compared to the first visit. Thus, the observation of I in the choroid may be useful for monitoring PD.

In this study, we investigated the follow-up of the retina and choroid in patients with PD. We hypothesized that the duration of PD would reduce the thickness of the retina and choroid. However, the results obtained from seven patients with PD indicate that the length of the disease duration period is not necessarily the factor that reduces the thickness of the retina and choroid. This result indicates that the PD duration may not be correlated with the amount of decrease in the choroid.

This study has several limitations. Although significant differences in the retina and choroid were observed, the number of participants was too small to allow us to draw definitive conclusions. Additionally, the retina was evaluated in the macula, and the choroid was evaluated only in the subfoveal tomographic image. Therefore, it may be different from the evaluation of the entire eye. Moreover, the correlation of the retina and choroid with severity classifications from Hoehn and Yahr could not be investigated. Sari et al. [23] reported that the retinal thickness was correlated with the severity classification of Hoehn and Yahr. Contrastingly, Eraslan et al. [20] reported that the retinal thickness was decreased but not correlated with the severity classification for Hoehn and Yahr. In this study, the severity of all patients was stage III at the first visit. After 3 years, two subjects had stage IV severity, but more cases need to be considered. In this study, choroidal binarization reduced I in patients with PD, and we focused on melanocytes. However, there are various cells other than melanocytes in the interstitial region, so we cannot exclude the possibility of other influencing factors on the current results; therefore, more studies and histological analyses are required to corroborate our findings. Moreover, the changes in the retina and choroid of patients with PD after 3 years were discussed considering the length of the disease period; however, to clarify this, it is necessary to conduct a comparative study in groups divided by the disease duration. All patients with PD were treated with levodopa. Levodopa treatment does not affect the retina [24]; however, it has been suggested to affect choroidal vessels [25]. Therefore, the effect of the therapeutic drug on the change in the choroidal vascular region cannot be ruled out in this study. However, at 3-years follow-up, there was no change in the choroidal luminal area despite continuing levodopa treatment, and a decrease in the interstitial area was observed. Therefore, it is considered that the choroid of patients with PD is affected not only by the therapeutic drug but also by neurodegeneration. With only a few reports on the effects of levodopa on the retina and choroid, additional research is needed to confirm this.

Evaluation of the retina and choroid using SS-OCT may serve as a biomarker for the assessment of central nervous degenerative diseases [26, 27]. The current findings suggest that autonomic dysfunction and neurodegeneration in PD result in structural changes in the retina and choroid.

## Declarations

### Availability of data and material

The datasets generated and/or analyzed during the current study are available from the corresponding author upon reasonable request.
